# The role of qualification and quality management in the prescription of antipsychotics and potentially inappropriate medication (PIM) in nursing home residents in Germany: results of the HIOPP-3-iTBX study

**DOI:** 10.1007/s40520-023-02513-9

**Published:** 2023-08-07

**Authors:** Regina Stolz, Olaf Krause, Ulrike Junius-Walker, Petra Thürmann, Angela Fuchs, Stefan Wilm, Anja Wollny, Franziska Rebentisch, Birgitt Wiese, Stefanie Joos, Hannah Haumann

**Affiliations:** 1 Institute for General Practice and Interprofessional Care, Medical Faculty, University Tübingen, Osianderstr. 5, 72076 Tübingen, Germany; 2 Institute for General Practice and Palliative Care, Hannover Medical School, Carl-Neuberg-Str. 1, 30625 Hannover, Germany; 3 Chair of Clinical Pharmacology, Faculty of Health, University Witten/Herdecke, Helios University Hospital Wuppertal University Witten/Herdecke, Heusnerstr. 40, 42283 Wuppertal, Germany; 4 Institute of General Practice, Medical Faculty, Centre for Health and Society, Heinrich-Heine University Düsseldorf, Moorenstr. 5, 40225 Düsseldorf, Germany; 5 Institute of General Practice, University Medical Center Rostock, Doberaner Strasse 142, P.O. Box 108880, 18057 Rostock, Germany

**Keywords:** Medication safety, Interprofessional care, Nursing home, Quality management, Antipsychotics, Potentially inappropriate medication

## Abstract

**Background:**

Nursing home residents (NHR) show high rates of polypharmacy. The HIOPP-3-iTBX study is the first cRCT on medication optimization in nursing homes (NH) in Germany. The intervention did not result in a reduction of PIM and/or antipsychotics. This analysis looks at structure quality in the HIOPP-3-iTBX study participants.

**Aims:**

Evaluation of structure quality as part of a cluster-randomized controlled intervention study.

**Methods:**

Structure quality in multiprofessional teams from *n* = 44 NH (*n* = 44 NH directors, *n* = 91 family doctors (FD), and *n* = 52 pharmacies with *n* = 62 pharmacists) was assessed using self-designed questionnaires at baseline. Main aspects of the questionnaires related to the qualification of participants, quality management, the medication process and size of the facilities. All completed questionnaires were included. number of PIM/antipsychotics was drawn from the baseline medication analysis in 692 NHR. Data were analyzed by descriptive statistics and mixed model logistic regression.

**Results:**

The presence of a nurse with one of the additional qualifications *pain nurse* or *Zertifiziertes Curriculum (Zercur) Geriatrie* in the participating NH was associated with a lower risk for the prescription of PIM/antipsychotics. No association between any characteristic in the other participants at baseline was observed.

**Conclusions and discussion:**

The results support the known role of nursing qualification in the quality and safety of care. Further studies need to look more closely at how use is made of the additional qualifications within the multiprofessional teams. Perspectively, the results can contribute to the development of quality standards in NH in Germany.

**Supplementary Information:**

The online version contains supplementary material available at 10.1007/s40520-023-02513-9.

## Background

Aging societies worldwide are associated with an increase in the rate of polypharmacy, which is frequently characterized by the concurrent use of five or more medications [1]. The rate of polypharmacy greatly varies from 4% to about 96.5% depending on the age group, definition, healthcare setting, and region studied [2]. Nursing home residents (NHR) show particularly high rates of polypharmacy when compared to older people living at home in international studies, as well as in Germany [3–6]. Polypharmacy is associated with numerous clinical consequences such as higher mortality and an increased risk for adverse drug events (ADEs), falls, and hospitalization [2, 7]. Among the elderly, polypharmacy also increases the risk of receiving potentially inappropriate medications (PIM). PIMs are described as medications which should be avoided in older adults, as detailed on the Beers list of the American Geriatric Society [8] and adapted to the drug markets and prescribing preferences of other countries, e.g., Japan [9], France [10] and Germany [11]. The use of drugs on PIM-lists is associated with a higher risk of ADEs [12, 13] and also mortality [14].

The risk of receiving PIM is also higher in NHR compared to older people living in the community [12]. In addition, PIM are associated with a higher risk of ADEs [15, 16]. Furthermore, approximately 50% of NHR receive antipsychotics which have been associated with cardiovascular events, hip-fractures, and increased all-cause mortality in that group [17–19].

Drug management in NHR is a complex interprofessional six-step process that involves the collaboration of NHR (or their relatives/representatives), physicians (mostly family doctors), nurses, and pharmacists [20]. The six steps of prescribing and discontinuing medication are: evaluation of medication regime, coordination with NHR, prescription proposal, and communication, drug dispense and delivery, drug use, and monitoring/follow-up (see also Fig. 1).

NHR are more likely to get a PIM prescription when collaboration is unstructured [3, 21]. It is known that quality standards such as implemented concepts for person-centered care in nursing homes (NH), concepts for dealing with behavior experienced as challenging and valid standards for pain management influence the number of antipsychotics prescribed [22, 23]. In Germany, there are no interprofessional standards implemented regarding the medication process in NHR. However, according to the German Pharmacy Practice Legislation (§12a ApoG), the delivery of drugs to a NH has to be regulated by a contract between the pharmacy and the NH, and the owner of the pharmacy is obliged to inform nurses and patients about appropriate drug use and risks. In addition, many family doctors (FD) apply quality management standards in their practices. In general, it is known that structured medication management is associated with medication safety in primary care [24]. The German clinical guideline on multimedication in family medicine recommends a structured, planned, and, as far as possible, committing communication between FD, pharmacist, and NH [20] in the care for NHR. Previous studies in German nursing homes show that this recommendation is not implemented in all nursing homes and everyday practice [3, 4]. Since October 2019, an indicator-based quality and audit system for NH has been introduced in Germany. The system includes nursing-sensitive outcome-based indicators of six quality areas, one of which contains the topics of drug therapy and pain management [25]. Nursing-sensitive indicators are defined as criteria for changes in health status that can be affected by nurses directly [26]. In the United States, the percentage of NHR receiving antipsychotic medication is an implemented quality criteria in NH already [27]. A systematic review by Oner et al. showed that there is an inverse relationship between medication administration errors and the proportion of registered nurses in NH, the working environment, and work during night shifts [26]. No significant relationship was shown between ADEs and other nursing-sensitive variables. So far, there are no data available on the relationship between the association of nursing qualification and medication safety and the prescription of PIM/ antipsychotics in particular in German NH.

According to Donabedian, quality of care comprises two main aspects: 1. technical care—meaning the extent to which the provided care maximizes the health benefits of an individual without increasing risk (a valuation that is to be shared between the caregivers and the patient), and 2. the psychosocial interaction between caregiver and receiver [28]. In this concept, it is important to consider three domains of quality: 1. structure quality (comprising quality of the structural factors that affect the performance of care), 2. process quality or the quality of the direct care that the staff provides, and 3. outcome quality including the impact for the individual patient or the health care service outcome of a population.

The HIOPP-3-iTBX study (HIOPP, primary care initiative to optimize patient safety in polypharmacy; iTBX, interprofessional toolbox) is the first cRCT on medication optimization in NH in Germany [29]. In contrast to previous open observational studies, the complex intervention in the HIOPP-3-iTBX study did not lead to a reduction of PIM and/or antipsychotic drugs, nor any improvement in the overall health status of the NHR studied [3, 4]. In line with the model of Donabedian and the new audit guideline for NH in Germany, the analysis of HIOPP-3-iTBX so far has focused on outcome quality [29, 30]. In the present work, the focus of the analysis focuses on aspects of structure quality. The aim of this study, therefore, is to 1. describe structures in the participating professions and institutions, 2. analyze the potential relationship between structures identified and the number of PIM and/or antipsychotic drugs taken by the participating patients in the baseline data of the HIOPP-3-iTBX study.

## Methods

### HIOPP-3-iTBX study

Between May 2018 and July 2019, a cRCT was conducted in 44 German NH at four study centers (the Institutes for General Practice in Düsseldorf, Hannover, Rostock, and Tübingen; Germany) [29]. Each nursing home formed a cluster in which a multiprofessional team was formed including nurses, pharmacists, and FD serving NHR in that particular NH. The inclusion criterion for the NHR was age ≥ 65 years. The complex intervention addressed all involved professions and comprised a medication review, training of the participants, the implementation of a toolbox, and change management measures. The primary endpoint of the study was the proportion of NHR with at least one PIM and/or at least two antipsychotic drugs 6 months after the intervention [29, 31].

### Analyzed data

As a part of the project evaluation, questionnaires addressing structural aspects in HIOPP-3-iTBX study participating FD (*n* = 91), pharmacists (*n* = 62), and NH (*n* = 44) were distributed at baseline. The questionnaires were self-developed within the study team in a consensus process, then pre-tested using cognitive interviews in the analyzed population and subsequently adapted. Main aspects of the questionnaires related to the qualification of participants, work experience, quality management, regulations on the medication process, number of employees in participating facilities, and size of NH/NHR. The number of PIM/antipsychotics taken by participating patients was drawn from the current medication plan of NHR at baseline. The relevant nursing qualifications for the present analysis were: 1. Zercur Geriatrie: developed by the German Geriatrics Association, is an advanced training course that aims to impart interdisciplinary knowledge in the field of geriatrics to nursing professionals, with aspects, such as ethics, palliative care, medication, dementia/depression, and pain. The curriculum comprises up to 180 h (basic qualification) and a total of 520 h of training to become a “Zercur Geriatrics Nursing Specialist”; 2. Pain nurses: nursing professionals with certified advanced training on pain and its management that comprises up to 200 h of training. The curriculum is based on the Pain Therapy Curriculum for Integrated Training and Continuing Education in Nursing of the German Pain Society (DGSS)—Section of the International Association for the Study of Pain (IASP); 3. Geriatric psychiatry: an additional qualification offered in varying amounts of training hours (from about 100 h (basic qualification) up to about 1000 h (advanced specialized training), most curricula include approx. 600 h).

In the present analysis, all completed questionnaires by participating nursing home directors, FDs, and pharmacists at baseline were included.

### Statistical methods

Descriptive results are presented as absolute frequencies and percentages or as mean ± standard deviation (SD). To evaluate the association of different factors with the proportion of NHR receiving a PIM/antipsychotic at baseline, a mixed model logistic regression was applied. In the logistic regression model data from *n* = 786 of the *n* = 787 NHR in the baseline examination (99.8%) were included. Accounting for the cluster effect of the NH, their identification code was included as a random effect in the model. The fixed factors in the model were: additional qualifications in nurses (gerontopsychiatry, pain nurse, palliative care, wound expert, *Zertifiziertes Curriculum (Zercur) Geriatrie* and others), the presence of a concept for dealing with challenging behavior in NHR (yes/no), the presence of a concept for avoiding measures that deprive patients of their freedom (yes/no), number of drugs and number of comorbidities. Age, gender, and dementia status of the NHR were included as adjusting factors. All analyses were performed with SPSS® Version 27 and StataSE Version 16.

## Results

In the HIOPP-3 iTBX study, *n* = 44 NH directors, *n* = 91 FDs, and *n* = 52 pharmacies with *n* = 62 pharmacists participated. Structure quality data from all FDs and NHs provided by their directors, and *n* = 50 (95%) pharmacies and *n* = 59 pharmacists (95%) was analyzed. Descriptive data of NHR and NH can be found in Tables 1 and 2, data of FDs and pharmacists in Tables 4 and 5 in the supplement. Association of potential influencing variables of NHR, NH structures, and the prescription of PIM/antipsychotics can be found in Table 3. Additional qualifications of nursing staff were asked about as characteristics of the NH (see Table 2). On average, between 2 and 3 of the five additional qualifications surveyed were available in the 44 participating NH. Most often were palliative care (*n* = 31), gerontopsychiatry (*n* = 26), and wound management (*n* = 25). Pain nurse qualification was available in 7 NH and Zercur Geriatrie in 2 NH.

**Table 1:** Sociodemographic characteristics of nursing home residents (NHR, *n* = 787) at baseline (T0)

Ø Age in years (SD)	84.3 (± 7.9)
Sex (female, %)	73.8 (*n* = 581)
NHR with legal guardianship, %	35.5 (*n* = 254)1
NHR educational status^a^, %	
Low	45.4 (*n* = 357)
Medium	30.5 (*n* = 240)
High	7.5 (*n* = 59)
Not specified	16.6 (*n* = 131)
Nursing home stay in months (median, IQA)	29 (13–53)
NHR care level, %	
None	0.4 (*n* = 3)
1 and 2	22.0 (*n* = 173)
3 and 4	64.7 (*n* = 508)
5	12.6 (*n* = 99)
Not specified	0.3 (*n* = 2)
Ø Number of diagnoses (SD)	7.4 (± 3.5)
Barthel Index NHR (ICD-Code U50.0–50), %	
None, mild (U50.0–10)	17.5 (*n* = 137)
Moderate, moderately severe (U50.20–30)	44.4 (*n* = 348)
Severe, very severe (U50.40–50)	38.1 (*n* = 299)
NHR with sign of dementia^b^, %	64.5 (*n* = 507)
Ø number of drugs prescribed (SD)	11.1 (± 4.5)
NHR with ≥ 1 PIM and/or ≥ 2 antipsychotics, %	41.3 (*n* = 325)

*NHR* nursing home resident; *PIM* potentially inadequate medication; Ø average; *SD* standard deviation, % percentage NHR

^a^Educational status according to CASMIN[32]

^b^Sign of dementia: identified by the nursing home or family physician

**Table 2:** Characteristics of nursing homes (NH, *n* = 44)

NH provider (%)	
Private	29.5 (*n* = 13)
Public (e.g., Municipal sponsorship)	6.8 (*n* = 3)
Non-profit (e.g., Red cross, welfare organization)	63.6 (*n* = 28)
Qualification of NH director (%)	
Advanced training	34.1 (*n* = 15)
Bachelor degree	2.3 (*n* = 1)
Diploma degree	36.4 (*n* = 16)
Master degree	11.4 (*n* = 2)
Other	4.5 (*n* = 2)
Not specified	11.4 (*n* = 5)
Qualification head of nursing service (%)	
Advanced training	88.6 (*n* = 39)
Bachelor degree	2.3 8(*n* = 1)
Diploma degree	4.5 (*n* = 2)
Master degree	2.3 (*n* = 1)
Other	2.3 (*n* = 1)
Type of record of care (%)	
Paper-based	11.4 (*n* = 5)
Electronic	63.6 (*n* = 28)
Combination of paper-based and electronic	25.0 (*n* = 11)
Number of pharmacies associated with NH (%)	
1	75.0 (*n* = 33)
2	20.5 (*n* = 9)
3	4.5 (*n* = 2)
Ø Family doctors per NH (SD)	9.76 (± 6.55). min/max 1/35
Ø Rate of specialized staff (%, SD)	53.02 (± 6.02). min/max 33/70
Additional qualification of nursing staff (%)	
Geriatric psychiatry	59.1 (*n* = 26)
Pain nurse	15.9 (*n* = 7)
Palliative care	70.5 (*n* = 31)
Wound management	56.8 (*n* = 25)
Zercur Geriatrie	4.5 (*n* = 2)
Other	40.9 (*n* = 18)
Concept challenging behavior among NHR (%)	
None	34.1 (*n* = 15)
Based on the framework recommendation on dementia^a^	36.4 (*n* = 16)
Other	20.5 (*n* = 9)
Yes, not specified	4.5 (*n* = 2)
Not specified	4.5 (*n* = 2)
Concept avoidance of measures involving deprivation of liberty (%)	
None	13.6 (*n* = 6)
Redufix^b^	6.8 (*n* = 3)
Werdenfelser way^c^	50.0 (*n* = 22)
Other	25.0 (*n* = 11)
Yes, not specified	4.5 (*n* = 2)
Quality management representative (%)	95.5 (*n* = 42)
Quality management system in use (%)	
None	2.3 (*n* = 1)
DIN/ISO	29.5 (*n* = 13)
Other	38.6 (*n* = 17)
Yes, not specified	20.5 (*n* = 9)
Not specified	9.1 (*n* = 4)

*SD* standard deviation; *NHR* nursing home resident; *NH* nursing home; *min* minimum; *max* maximum; Ø average; % percentage of NH

^a^Ref.[33]

^b^Ref.[34]

^c^Ref.[35]

**Table 3:** Association of potential influencing variables of NHR, NH structures, and the prescription of PIM/antipsychotics in *n* = 786 NHR, results of a mixed model logistic regression

Variable (reference)	OR (95% CI)	*P* value
Sociodemographics of participating patients		
Age	0.99 (0.97–1.01)	0,453
Female sex	1.20 (0.82–1.76	0,351
Dementia diagnosed	1.98 (1.40–2.80)	0,000
Additional qualification of nursing staff		
Gerontopsychiatry	1.35 (0.85–2.13)	0,200
Pain nurse	0.55 (0.33–0.91)	0,020
Palliative care	1.07 (0.70–1.65)	0,752
Wound manager	1.28 (0.86–1.92)	0,230
Zercur geriatrics	0.30 (0.12–0.71)	0,006
Other	0.80 (0.52–1.23)	0,302
Nursing concepts regarding		
Challenging behavior among nursing home residents	0.83 (0.54–1.29)	0,412
Avoidance of measures involving deprivation of liberty	1.21 (0.67–2.19)	0,527
Further aspects		
Number of drugs	1.15 (1.10–1.20)	0,000
Number of comorbidities	0.98 (0.93–1.03)	0,338

*OR* odds ratio; *95% CI* 95% confidence interval

Regression analysis showed that at baseline a higher number of PIM/antipsychotics was associated with the presence of dementia in the NHR (OR: 1.70; 95% CI 1.23–2.34; *p* = 0.001). On the NHR level analysis the presence of a nurse with one of the additional qualifications *pain nurse* or *Zertifiziertes Curriculum (Zercur) Geriatrie* in the participating NH showed a statistically significant association to a lower risk for the prescription of PIM/antipsychotics (*pain nurse* OR: 0.59; 95% CI 0.35–0.96, *p* = 0.044 Z*ercur*; OR 0.21; 95% CI 0.09–0.51; *p* = 0.001). No association between any characteristic in FDs and pharmacies/pharmacists and the number of PIM/antipsychotics prescribed at baseline was observed.

## Discussion

To our knowledge, HIOPP-3-iTBX is the first randomized study to investigate structures, processes, and outcomes of an interprofessional medication management in NH in Germany. The complex intervention in HIOPP-3-iTBX did not lead to a reduction of PIM and/or antipsychotics in the participating NHR [29]. In this analysis, the focus is on aspects of structure quality and their possible effects on the prescription of PIM/antipsychotics in NHR at baseline of the HIOPP-3-iTBX study. In our study, the structural parameters collected from FD and pharmacists had no effects. We focused, therefore, on the qualification of nurses rather than the involvement of pharmacists, of which the latter has been shown as a success factor in other studies [36, 37].

The analysis showed that dementia in NHR and number of drugs were associated with a higher risk for the prescription of PIM/antipsychotics. Specific additional nursing qualifications regarding pain and geriatrics (*ZERCUR GERIATRIE*) were associated with a lower risk for the prescription of PIM/antipsychotics. No further associations to any of the study participant's characteristics (NHR, FD, and pharmacists) or their qualifications were found. The results show that the qualification of the nursing staff influences medication safety in the studied NH.

Previous work shows that higher qualification of nursing professionals is associated with improved patient outcomes [38, 39]. Regression analysis did not show a significant correlation between nurses' additional qualification in gerontopsychiatry and a higher risk for the prescription of PIM/antipsychotics to participating patients. Currently, it is not obligatory to have nurses with either one of the described advanced training in teams in NH in Germany. However, pain is a relevant issue in geriatrics. Inadequate pain management is known to be a frequent quality problem in NH leading to agitation and the prescription of antipsychotics as a consequence [40, 41]. Furthermore, the experience of pain is commonly associated with cognitive impairment [42–47]. Appropriate analgesia may reduce pain and its associated cognitive impairment. The lack of sensitivity of the medical staff in recognizing pain based on non-verbal cues/pain-related behavior in people suffering from cognitive impairment is a known cause of suboptimal pain management in this population [48, 49]. Untreated pain is considered to be one of the causes of challenging behavior, such as agitation or aggression in people with dementia [50]. On the other hand, in some cases, cognitive impairment may worsen under analgesic medication [51, 52]. Interprofessional collaboration in medication management, therefore, is necessary for the management of pain in NHR. Nurses can make a significant contribution here. Namely, the contribution comprises managing and monitoring patients’ medication and possible ADEs. Interprofessional medication management with the participation of FD (and possibly other specialized doctors), pharmacists, and nurses comprises the aspects named before. A model of an interprofessional medication process is shown in Fig. 1. Ideally, nurses are involved in all of the six steps. For example, nurses play an important role in tapering antipsychotics. The process of tapering, e.g., antipsychotics, should take place over a period of 2–8 weeks and requires good observation of the reaction of the NHR by nurses [53].

However, interprofessional cooperation means more than just the sum of tasks distributed among different disciplines. It is a model of collaboration between different healthcare providers in which the different professional groups have an awareness of the competencies and different roles of the other team members and are aware of a common goal for the patient (patient-centeredness) [54]. Both advanced trainings Zercur geriatrie and pain nurse were in our analysis found to be associated with a lower rate of PIM/antipsychotics in the NHR. Both trainings also comprise the knowledge and competencies named earlier, including aspects of interprofessional collaboration. Studies so far show that staff level and staff training have an impact on the reduction of inappropriate prescribing of antipsychotics in people with dementia [55]. One study showed that it is often nurses that initiate the prescription of antipsychotics in NHR [41]. Furthermore, it is known from the literature that applying this nursing-specific knowledge improves patient outcomes, including pain, and aggression. However, little is known about the exact relationships between certain skill levels, the nurse staffing ratio, and patient outcomes in NH [26]. Our results further support the initiatives to train nurses in their role in medication management specifically [56]. In Germany, a structured interprofessional study program on drug therapy safety needs analysis and design of a model curriculum (SINA) was developed [57]. It will be of interest to evaluate how this interprofessional training on drug therapy affects medication safety in nursing home care and the contribution of the different professions, as one focus of SINA is the role of pharmacists within interprofessional teams.

To summarize, our results emphasize the importance of nursing quality and qualifications for patient safety in NH.

## Limitation

Only a few nurses in the HIOPP-3-iTBX study had one of the additional qualifications Pain Nurse (*n* = 7) or Zercur Geriatrie (*n* = 2); however, on the NHR-level analysis, these nursing qualifications and the rate of NHR with PIM/antipsychotics show a statistically significant association. As a limitation, it is to be mentioned that the exact number of hours of the advanced training course present in the different nurses was not asked for. Based on the present data, it is unclear why the additional qualification gerontopsychiatry is not associated with the rate of PIM/ antipsychotics. However, in four of the seven NH with pain nurses there was a nurse with additional qualification in gerontopsychiatry, just like in one of the two NH with a nurse qualified according to Zercur Geriatrics.

Data from the HIOPP-3-iTBX study does not allow for a description more detailed how the trained nursing professionals apply their knowledge in daily care. In addition, there is no data on whether the presence of nurses with additional qualifications changes the medication processes related to the prescription of PIM/ antipsychotics. Possible hypotheses are that the NH with specialized nurses are more innovative overall with possible side-effects such as more in-house training and case discussions or different attitudes towards interprofessional cooperation. This aspect needs to be investigated in further studies. Therefore, important information to undermine the findings is lacking. A further limitation could be an unintended selection bias: it is hard to rule out that NH, pharmacists, and FDs with a high interest in the topic participated in the study.

## Conclusions

Medication management in NH is an interprofessional and complex task. The results of this analysis of the HIOPP-3-iTBX study support the known role of nursing qualification in the quality and safety of care. The analysis showed that advanced training in the field of pain and geriatrics in nursing professionals was associated with lower rates of PIM/antipsychotics in NHR at baseline of the HIOPP-3-iTBX study [20].

Based on the results, further studies should examine the effect of additional nursing qualifications in German NH more closely (e.g., number of qualified staff in NH, extent of qualification, collaboration/role within nursing team). Moreover, further studies are important to evaluate the role of advanced trained nurses in the medication process with a focus on the initiation of a prescription and its evaluation regarding PIMs and antipsychotics, especially in nursing teams in Germany. As quality standards for German NH are under development, the present and future studies can perspectively contribute to their development and introduction.

### Supplementary Information

Below is the link to the electronic supplementary material.

Supplementary file1 (DOCX 16 KB)

Supplementary file2 (DOCX 15 KB)

Supplementary file3 (JPG 149 KB)
