# Supplementary material for: The role of qualification and quality management in the prescription of antipsychotics and potentially inappropriate medication (PIM) in nursing home residents in Germany: results of the HIOPP-3-iTBX study
Source: Aging Clin Exp Res. 2023 Aug 7;35(10):2227–35. doi: 10.1007/s40520-023-02513-9 (PMC10520111; doi:10.1007/s40520-023-02513-9)
Supplement: Supplementary file 1 — Supplementary file1 (DOCX 16 KB) [file 40520_2023_2513_MOESM1_ESM.docx]

**Table 4 Characteristics of participating family doctors (n=92)**

| **Male Sex, %** | 60,9 |
| --- | --- |
| **Age (SD)** | 54,7 (±8,9), min/max 40/71 |
| **Ø work experience in years (SD)** | 20,1 (±20,59), min/max 1/ 42 |
| **Specialty training (%)**   - Family physician - Internal medicine - Medical practitioner - other - not specified | 58,1  32,6  2,2  5,5  1,2 |
| **Additional qualification (multiple answer set) (%)**   - Palliative care - Emergency medicine - Quality management - Rehabilitation - Psychotherapy - Not specified | 25  23,9  5,4  5,4  5,4  31,5 |
| **Not an academic practice (%)** | 45,7 |
| **Type of practice, %**   - Solo practice - Praxisgemeinschaft - Group practice - Regional?? group practice - Medical care center - Sonstige - Not specified | 29,3  6,5  46,7  2,2  1,1  3,3  4,3 |
| **Ø NH per practice (SD)** | 4,27 (±2,54), min/max 1/15 |
| **Ø number of patients per quarter (%)**   - 501-1000 - 1001-1500 - 1501-2000 - >2000 - Not specified | 10,9  15,2  20,7  34,8  9,8 |
| **Inhabitants site of practice %**   - <5000 - 5001-20000 - 20001-100000 - >100000 - Not specified | 3,3  20,7  21,7  51,1  2,2 |
| **Quality management system installed %** | 68,5 |
| **SOP medication process implemented, %**  **Type of SOP (multiple answer set), %**   - SOP allergies - SOP Documentation of ADR - SOP Annual Medication check in risk patients - SOP medication monitoring - recallsystem - other - not specified | 68,5  51,1  30,4  26,1  28,3  22,8  4,3  1,1 |
| SD: standard deviation; min: minimum; max: maximum; SOP: standard operation procedure | |
