# Supplementary material for: The role of qualification and quality management in the prescription of antipsychotics and potentially inappropriate medication (PIM) in nursing home residents in Germany: results of the HIOPP-3-iTBX study
Source: Aging Clin Exp Res. 2023 Aug 7;35(10):2227–35. doi: 10.1007/s40520-023-02513-9 (PMC10520111; doi:10.1007/s40520-023-02513-9)
Supplement: Supplementary file 2 — Supplementary file2 (DOCX 15 KB) [file 40520_2023_2513_MOESM2_ESM.docx]

**Table 5: Characteristics of participating pharmacies (n=50) and pharmacists (n=59)**

| **Ø number of NHR per pharmacy (SD)** | 212,42 (±188,84), min/max: 30/1000 |
| --- | --- |
| **Ø number of visits in nursing home (SD)**   - numberl/day - number/week - number/month - number/year | 1,5 (±0.95), min/max 1/5  5,44 (±3,81), min/max 2/15  1,5 (±0,71), min/max 1/2  3,75 (±0,89), min/max 2/5 |
| **Female Sex, %** | 61 |
| **Ø age (SD)** | 44,83 (±12,21), min/max 26/68 |
| **Ø years work experience (SD)** | 18,16 (±12,29), min/max 1/48 |
| **Other qualifications (multiple anwer set), %**   - geriatric pharmacy - curriculum by federal medical association (BAK) - other - not specified | 6,8  1,7  33,9  59,3 |
| **Ø distance between NH and pharmacy in km (SD)** | 2,98 (±4,417), min/max 0/25 |
| **No pharmacists in charge of nursing homes; %** | 52 |
| **Quality management in pharmacy %** | 82% |
| Ø: mean; NHR: nursing home resident; SD: standard deviation; min: minimum; max: maximum; BAK: Bundesärztekammer | |
