# Supplementary figures and images for: The role of qualification and quality management in the prescription of antipsychotics and potentially inappropriate medication (PIM) in nursing home residents in Germany: results of the HIOPP-3-iTBX study
Source: Aging Clin Exp Res. 2023 Aug 7;35(10):2227–35. doi: 10.1007/s40520-023-02513-9 (PMC10520111; doi:10.1007/s40520-023-02513-9)

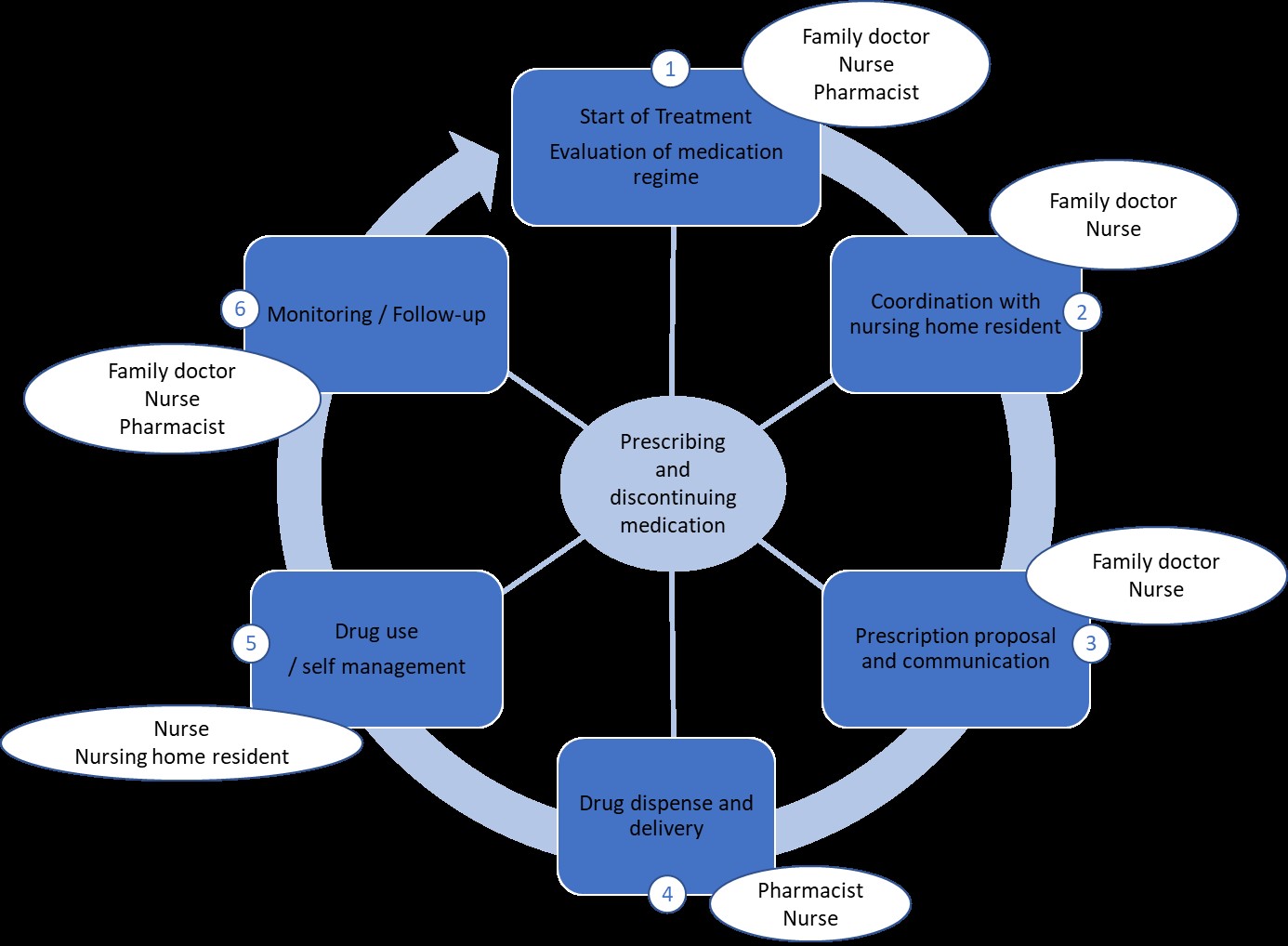

Supplement: Supplementary file 3 — Supplementary file3 (JPG 149 KB) [file 40520_2023_2513_MOESM3_ESM.jpg]
